# Nutrition and Cardiovascular Diseases

**DOI:** 10.3390/nu14010094

**Published:** 2021-12-27

**Authors:** Yoshihiro Fukumoto

**Affiliations:** Division of Cardiovascular Medicine, Department of Internal Medicine, Kurume University School of Medicine, Kurume 830-0011, Japan; fukumoto_yoshihiro@med.kurume-u.ac.jp; Tel.: +81-942-31-7562; Fax: +81-942-33-6509

Unhealthy food intake and insufficient physical activities are related with obesity or life-style diseases, which can cause cardiovascular diseases, finally leading to death [1,2]. First, to prevent the progression of obesity or life-style diseases, nutrition and exercise are the most important issues. However, many people are not aware of their importance. Second, after the development of obesity or life-style diseases, nutrition and exercise control with appropriate medical therapies are required. Still, many patients do not recognize this. Third, after the cardiovascular diseases, nutrition and exercise with optimal medical and/or interventional therapies are required. However, some patients are not able to control their food intake and physical activities. Finally, patients with end-stage cardiovascular diseases need their end-of-life discussion. In the current Special Issue of Nutrients, the importance of nutrition was reported before and after cardiovascular diseases development.

Regarding BEFORE cardiovascular diseases development, Wang et al. [3] have shown the strong evidence that daily milk intake among Japanese men was associated with delayed and lower risk of mortality from stroke (especially cerebral infarction, using data from the JACC study) as a good eating habit. The time before an event of stroke mortality occurred was slowed down among men who drank milk regularly. [3] Also in the aspect of healthy food, Zhu et al. [4] have demonstrated that their secondary analysis showed that long-term consumption of specific healthy plant foods including nuts, fruits, and vegetables improved weight management and cardiometabolic health, whereas adherence to an overall plant-based diets benefited weight management only, supporting the hypothesis that specific components in plant-based diets are important as well. As a good habit in a high-risk group, Alehagen et al. [5] have reported the relationship between D-dimer and a dietary supplement consisting of selenium and coenzyme Q10 in an elderly community-living population. They observed a significantly reduced cardiovascular mortality in the population with a high D-dimer level when given selenium and coenzyme Q10, as compared to those on placebo [5]. 

Conversely, unhealthy food issues were also reported. Ishida et al. [6] have revealed dose-response-positive associations between the number of accumulated unhealthy eating habits and the likelihood of obesity and central obesity in a general Japanese population, suggesting that modifying individual unhealthy eating habits and avoiding their accumulation might reduce the burden of obesity and central obesity [6]. As indicated in this article, healthcare professionals need to encourage those who have unhealthy eating habits to modify each of their habits individually, including avoidance of accumulating multiple unhealthy habits, in order to prevent cardiovascular diseases [6]. Further longitudinal studies should be performed to examine whether a causal relationship exists between the accumulation of unhealthy eating habits and the incidence of obesity or central obesity [6]. 

Regarding AFTER cardiovascular diseases development, the importance of nutrition in various types of cardiovascular diseases was reported. Matsuzaki et al. [7] have demonstrated that acquisition of effective behaviour modifications by long-term nutrition counselling, according to behavioural modification stages, was important for patients with cardiovascular diseases. 

In acute coronary syndrome (ACS), Okada et al. [8] have found a decreasing trend in eicosapentaenoic acid (EPA) and docosahexaenoic acid (DHA) levels, and EPA to arachidonic acid ratio in men from 2011 to 2019 without significant changes in low-density lipoprotein and high-density lipoprotein cholesterol levels, in which decreasing trend in EPA and DHA levels did not depend on age and was significant only in men in patients with ACS. Administration of a sufficient dose of n-3 polyunsaturated fatty acids may be effective in the secondary prevention of ACS, but further studies are needed to obtain robust evidence. Next, Creco A, et al. [9] have indicated that patients with ACS experience difficulties in achieving and mostly maintaining adequate levels of a healthy diet and physical activity over time, of which difficulties were modulated by environmental conditions, and most importantly, by psychological characteristics, suggesting how to perform tailor diet and physical activity interventions. They considered that tailoring should be aimed at promoting cognitive awareness of the risks associated with cardiovascular diseases recurrence. In fact, they showed that patients, who were more anxious, and therefore more concerned and somehow aware of their health, were more able to maintain healthy behaviour over time [9]. Cognitive awareness of the risks associated with cardiovascular diseases recurrence may be a useful tool to sustain patients’ capabilities to self-regulate their behaviours and to ameliorate lifestyle behaviour [9]. In atherosclerosis, blood glucose fluctuations are also important. Eto et al. [10] have indicated that patients with severe internal carotid artery siphon stenosis had higher blood glucose fluctuations as assessed with standard deviation, coefficient of variation, and mean amplitude of glycaemic excursions. Meanwhile, there were no significant differences in other vascular risk factors, such as hypertension, dyslipidaemia, mean blood glucose levels, haemoglobin A1c, and duration in years of type 2 diabetes mellitus, suggesting that glucose fluctuation can be a target of preventive therapies for intracranial artery stenosis and ischemic stroke [10]. 

In advanced cardiovascular diseases, low nutrition status is another problem. In this special issue, the correlation between nutrition status and acute myocardial infarction (AMI) with cancer was also reported. Itaya et al. [11] have indicated that the prevalence of cancer in AMI was 13%, and that worse nutrition status and renal dysfunction were associated with AMI with cancer, in which nutrition status was a major different characteristic from non-cancer. Further, they have developed formulas to predict the presence of cancer in AMI [11].

Next, in heart failure (HF), Kurunose et al. [12] have shown that patients with vitamin D supplementation had a lower in-hospital mortality for HF than patients without vitamin D supplementation in the propensity matched cohort. The causality should be tested in the future RCTs in specific population based on their study [12]. As also reported in ACS, Matsuo et al. [13] have found that low plasma DHA levels were significantly associated with an increase in all-cause death in patients with acute decompensated heart failure with preserved ejection fraction (HFpEF), independent of nutritional status, in which measurement of plasma DHA levels may be useful in identifying high-risk patients with HFpEF, and supplementation with DHA may be a potential therapeutic target in these patients. 

After HF development, adequate nutrition intake is important. Obata et al. [14] indicated that inadequate dietary calorie intake was independently associated with an increased risk of adverse clinical events including all-cause death and hospitalization due to worsening HF in stable patients with chronic HF. To meet the humanly end of HF life, nutritional care is quite important. Shibata et al. [15] have demonstrated that HF palliative care team activities were able to provide an opportunity to discuss end-of-life care with patients, reduce the burden of physical and mental symptoms, and shift the goals of end-of-life nutritional care to ensuring comfort and quality of life. 

In patients with end-stage kidney disease, HF is a serious condition characterized by decreased myocardial contractility and abnormal hemodynamic state [16]. It has been demonstrated that L-carnitine supplementation could improve clinical symptoms and cardiac function and decrease serum levels of B-type natriuretic peptide (BNP) and NT-proBNP in patients with chronic HF, [17] which might also improve cardiac function in hemodialysis patients [18], suggesting that L-carnitine treatment may have protective effects on HF in hemodialysis patients with carnitine deficiency. In the current issue, Sugiyama et al. [16] have reported that reducing L-carnitine administration for six months significantly decreased both plasma and red blood cell carnitine levels. Moreover, stopping L-carnitine increased plasma BNP levels. However, this stoppage did not influence cardiac function in hemodialysis patients [16].

Nutrition should be considered before and after cardiovascular development. According to these findings, we should pay more attention to preventive and therapeutic strategies for cardiovascular diseases.
